# Infectivity and stress tolerance traits affect community assembly of plant pathogenic fungi

**DOI:** 10.3389/fmicb.2023.1234724

**Published:** 2023-08-25

**Authors:** Soyoung Choi, Jung Wook Yang, Jung-Eun Kim, Hosung Jeon, Soobin Shin, Dayoun Wui, Lee Seul Kim, Byung Joo Kim, Hokyoung Son, Kyunghun Min

**Affiliations:** ^1^Department of Agricultural Biotechnology, Seoul National University, Seoul, Republic of Korea; ^2^Crop Cultivation and Environment Research Division, National Institute of Crop Science, Rural Development Administration, Suwon, Republic of Korea; ^3^Research Institute of Climate Change and Agriculture, National Institute of Horticultural and Herbal Science, Rural Development Administration, Jeju, Republic of Korea; ^4^Research Institute of Agriculture and Life Sciences, Seoul National University, Seoul, Republic of Korea

**Keywords:** *Fusarium graminearum*, *Fusarium asiaticum*, community assembly, high-throughput screening, competition assay

## Abstract

Understanding how ecological communities assemble is an urgent research priority. In this study, we used a community ecology approach to examine how ecological and evolutionary processes shape biodiversity patterns of plant pathogenic fungi, *Fusarium graminearum* and *F. asiaticum*. High-throughput screening revealed that the isolates had a wide range of phenotypic variation in stress tolerance traits. Net Relatedness Index (NRI) and Nearest Taxon Index (NTI) values were computed based on stress-tolerant distance matrices. Certain local regions exhibited positive values of NRI and NTI, indicating phenotypic clustering within the fungal communities. Competition assays of the pooled strains were conducted to investigate the cause of clustering. During stress conditions and wheat colonization, only a few strains dominated the fungal communities, resulting in reduced diversity. Overall, our findings support the modern coexistence theory that abiotic stress and competition lead to phenotypic similarities among coexisting organisms by excluding large, low-competitive clades. We suggest that agricultural environments and competition for host infection lead to locally clustered communities of plant pathogenic fungi in the field.

## 1. Introduction

A fundamental objective of ecology is to understand the mechanisms that mediate the assembly of natural communities (Stephen Brewer, 2000). There are various theories on how the assembly of a community occurs, and it is still a subject of debate (Webb et al., 2002; Mayfield and Levine, 2010; Goberna et al., 2014; Salazar et al., 2016; Sommer et al., 2017). Current theory predicts that the assembly of a community at the local level is determined by two primary ecological processes: the interaction between a species and its abiotic environment, and interactions among the species themselves (Gotzenberger et al., 2012). Fungi typically co-occur with evolutionarily related organisms more often than expected by chance, a process that results in clustering (Pellissier et al., 2014; Mykra et al., 2016; Chen et al., 2017). Clustering is a common pattern in ecological communities, in which species tend to co-occur with their evolutionary relatives more frequently than expected by chance. Classical community theory suggests that clustering results from the selection of taxa that share similar ecological traits (e.g., stress tolerance and morphology), allowing them to persist in a specific environment (Webb et al., 2002). These traits may be conserved across phylogenetic lineages, leading to related taxa clustering. Under the traditional framework, biotic interactions, particularly competition, drive the co-existence of organisms that are phenotypically distant, resulting in overdispersed patterns. This results from the competitive exclusion of ecologically similar organisms based on their niche similarities (Webb et al., 2002). Modern coexistence theory (Mayfield and Levine, 2010) has refined this view, arguing that competition can generate both phenotypic clustering when it operates through environmentally mediated differences in competitive abilities among entire clades. We postulate that this mechanism may operate in plant-pathogenic fungal communities, which are typically under environmental stresses and competition for host infections.

*Fusarium graminearum* and *F. asiaticum* are plant pathogenic fungi that cause Fusarium head blight (FHB) diseases in small grain cereals such as wheat, barley, and rice (van der Lee et al., 2015). In addition to the yield loss, they produce trichothecene mycotoxins, such as nivalenol (NIV), deoxynivalenol (DON), 15-acetyldeoxynivalenol (15-ADON), and 3-acetyldeoxynivalenol (3-ADON), as well as zearalenone (Desjardins, 2006). Mycotoxins threaten food security by causing toxicosis in livestock and humans, leading to economic losses and health risks. *F. graminearum* and *F. asiaticum* are closely related species that belong to *Fusarium graminearum* species complex (FGSC). A comparison of genome structure and gene content revealed a 93% overlap between the two species, which are also morphologically indistinguishable (O'Donnell et al., 2004). *F. asiaticum* was identified as the major species causing FHB outbreaks in cereal crops in the southern region of the Republic of Korea (Lee J. et al., 2009; Ahn et al., 2022). In terms of life cycle, both species are primarily soil-borne and overwinter as mycelia or spores in plant debris (Trail, 2009). In soil and plant debris, they are exposed to various environmental stresses such as water stress, oxidative stress, and agricultural chemical stress. They can infect crops at any stage of growth, but typically infect the heads of cereal plants during the flowering stage. Both species produce masses of spores that are spread by wind and rain, facilitating the spread of the disease to neighboring plants. The success of the fungal pathogens depends on their ability to successfully infect and spread within host plants. Another important factor in determining the success of the pathogens is their ability to persist as saprophytes on the dead plant debris long after the crop has been removed (Min et al., 2012). These factors can have a significant impact on the fungal community. Population genomics analyses of *F. graminearum* have been conducted, revealing a high level of genetic diversity and extensive recombination within the population (Talas and McDonald 2015; Talas et al., 2016).

In 2021, a FHB outbreak occurred in the southern region of the Republic of Korea (Kim, 2021; Supplementary Note S1). To determine the population structure of *F. graminearum* and *F. asiaticum*, 205 strains were isolated from diseased cereals crops at 18 sites. Stress tolerance of the strains was compared in 11 different conditions, using a high-throughput screening approach. Our competition assays revealed that the diversity of the fungal community decreased under chemical stress and host infection. In conclusion, we found that the selection for infectivity and stress tolerance traits leads to phenotypic clustering of fungal communities. These findings shed light on the mechanism driving the clustering of plant-pathogenic fungal communities and provide insights into the dynamics of plant-fungi interactions.

## 2. Materials and methods

### 2.1. *Fusarium graminearum* species complex isolation

**Figure 1:**
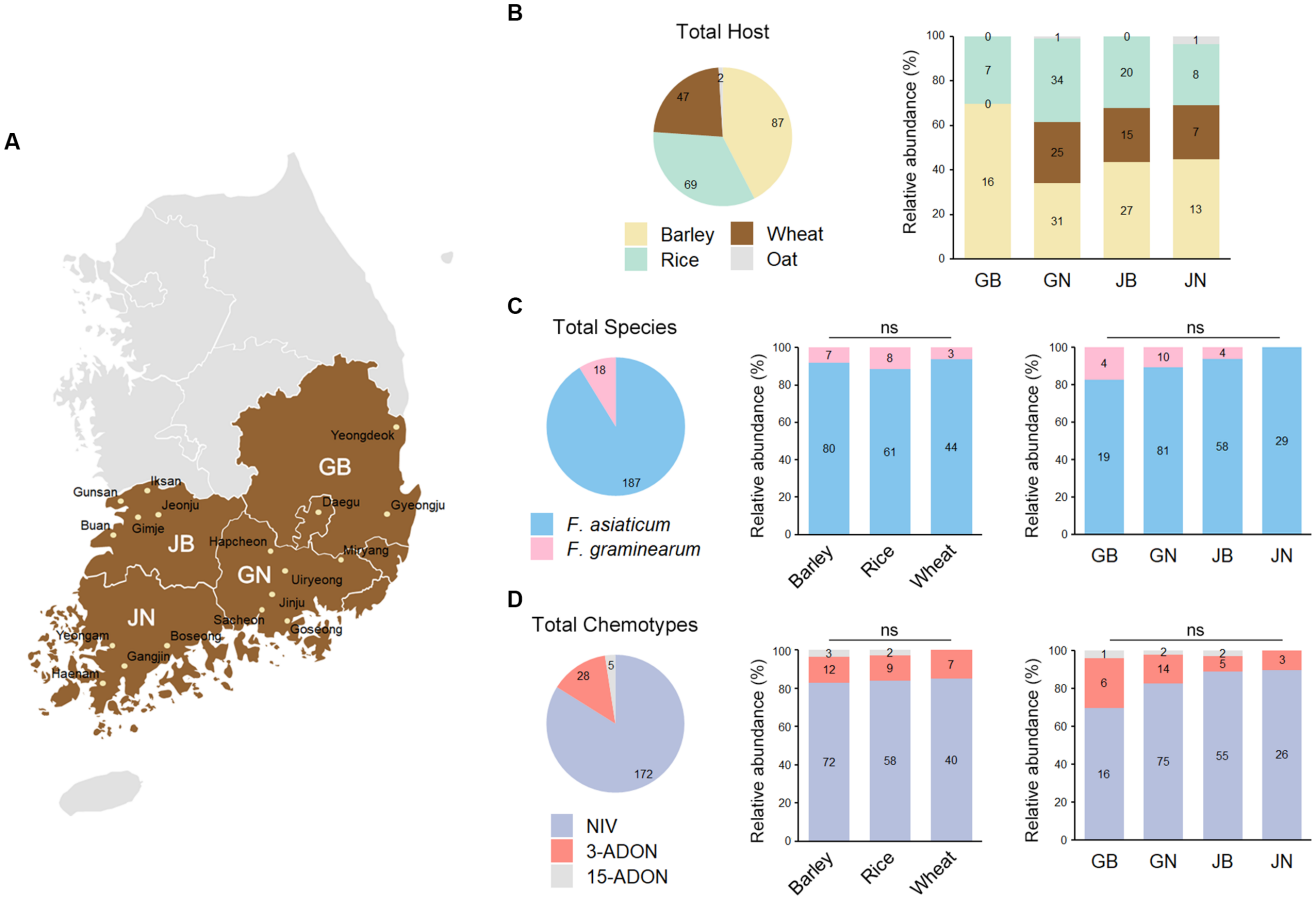
The distribution of *Fusarium graminearum* and *F. asiaticum* isolates in southern region of the Republic of Korea. **(A)** Map of the Republic of Korea indicating the 18 sampling sites in 4 southern provinces. GB, Gyeongsangbuk-do; GN, Gyeongsangnam-do; JB, Jeollabuk-do; JN, Jeollanam-do. **(B)** The pie chart illustrates the number of strains that have been isolated from the indicated hosts. The bar charts on the right show the percentage of hosts from the marked provinces, with the actual number of the hosts labeled inside the bars. **(C)** *F. asiaticum* represents 91% of the field isolates while *F. graminearum* accounts for the remaining 9%. The bar charts on the right show the percentage of *F. asiaticum* and *F. graminearum* strains isolated from the marked hosts or provinces, with the actual number of strains labeled inside the bars. There was no significant (ns) correlation observed between species and either host or location. **(D)** NIV chemotype represents 84% of the field isolates while DON chemotypes count for the remaining 16. There was not a significant correlation between chemotype and either host or location. NIV, nivalenol; 3-ADON, 3-acetyldeoxynivalenol; 15-ADON, 15-acetyldeoxynivalenol.

FGSC isolates were collected from different cereal fields in the southern region of the Republic of Korea in 2021. Cereal crops (barley, wheat, oat, and rice) with FHB symptoms were sampled from fields located in 18 cities/counties and 4 provinces (Figure 1A and Supplementary Data S1). The infected seeds were surface sterilized with 1% sodium hypochlorite and 70% ethanol solution. The seeds were placed onto potato dextrose agar (PDA, BD Difco, Franklin Lakes, New Jersey) plates and incubated for 3 days at 25°C. We isolated 205 FGSC strains based on morphological characteristics, such as colony pigmentation. Fungal strains were grown on PDA and stored in 20% glycerol solution at −80°C.

### 2.2. PCR amplification and sequencing, and phylogenetic analyses

Fungal isolates were cultured in liquid complete medium for 3  days at 25°C. Genomic DNA was extracted from lyophilized mycelia powder using a conventional Cetyltrimethylammonium Bromide (CTAB) method (Leslie and Summerell, 2006). The *TEF-1α* region was amplified by PCR using ExTaq polymerase in accordance with the manual (Takara Bio, Otsu, Japan). The EF-2 and FusF oligonucleotide pair was used for *TEF-1α* amplification (Supplementary Table S1). The oligonucleotides were synthesized at a commercial synthesis facility (Bioneer, Daejeon, Republic of Korea). The PCR products were purified using a commercial Kit (Intron, Seongnam, Republic of Korea) and submitted to the Bioneer PCR sequencing service. The oligonucleotide used for sequencing were the same as those used for amplification. The trimmed DNA sequences were BLAST-searched against the NCBI GenBank database for species identification (Supplementary Data S1, S2).

To determine the trichothecene chemotypes, we subjected the FGSC isolates to PCR using two primer sets. One set amplified the trichothecene 15-O-acetyltransferase (*TRI3*) gene, and the other set amplified the trichothecene efflux pump (*TRI12*) gene (Ward et al., 2002). Each set of primers include one common primer and three chemotype-specific primers for NIV, 3-ADON, and 15-ADON trichothecene chemotype, respectively (Supplementary Table S1). The amplified products were loaded onto 1.5% (w/v) agarose gel. The trichothecene chemotypes were predicted from the gel images based on the band size.

The *TEF-1α* sequences were used for the phylogenetic analysis of *F. graminearum* and *F. asiaticum*. Reference sequences of *F. graminearum* (NRRL 5883, NRRL 6394, NRRL 13383, NRRL 28063, NRRL 28336, NRRL 28439, and NRRL 29169), *F. asiaticum* (NRRL 6101, NRRL 13818, NRRL 26156, and NRRL 28720), and *F. pseudograminearum* strains (NRRL 28062, NRRL 28065, NRRL 28334, and NRRL 28338) were obtained from NCBI PopSet^1^ (O'Donnell et al., 2000). The DNA sequences were aligned using the Geneious Prime software and used to construct phylogenetic trees (Biomatters, Auckland, New Zealand). Maximum Likelihood analysis was performed utilizing the General Time Reversible model with Invariant sites and Gamma distribution (GTR + I + G). This model was selected due to its comprehensive approach, accounting for base substitution rates, invariant sites, and rate variations among sites (Abadi et al., 2019). The robustness of the obtained tree was evaluated by bootstrap analysis with 1,000 replicates (Pattengale et al., 2010).

### 2.3. High-throughput phenotypic screening

For conidia production, the fungal strains were incubated in carboxymethylcellulose medium (Cappellini and Peterson, 1965) for 7 days at 25°C in a shaking incubator at 0.314 rcf. The conidia were filtered through Miracloth (Millipore, Burlington, MA, United States) and resuspended in liquid complete medium to give 1 × 10^5^ conidia mL^−1^. We mixed 100 μL of conidia suspension with an equal volume of complete media supplemented with different chemicals on 96-well plates. Final concentration of chemicals in the cultures were 0.31 mM hydrogen peroxide, 1 M sorbitol, 1 M sodium chloride, 0.1 mg mL^−1^ sodium dodecyl sulfate, 0.05 μg mL^−1^ fludioxonil, 125 μg mL^−1^ iprodione, 0.625 μg mL^−1^ tebuconazole, 2 μg mL^−1^ tunicamycin, 1.25 mM dithiothreitol, 23.5 μL mL^−1^ hydrogen chloride (pH 2), and 5.3 μL mL^−1^ sodium hydroxide (pH 12). The cultures on the 96-well plates were incubated for 60 h at 25°C in plastic containers to prevent evaporation. There were two replicates for each condition. The OD_600_ value was measured every 12 h using a microplate reader a Synergy HTX microplate reader (BioTek, Winooski, VT, United States). The OD_600_ values at 48 h was used to compare stress tolerance. The absorbance of the medium alone was subtracted and the resulting values were averaged. To compare the growth of the strains under different stress conditions, the growth in complete medium was used as a reference point and the growth in stress conditions was normalized accordingly. The resulting values were used to calculate the Log2 fold difference from the average growth for heatmap analysis. Data analysis was performed using R software (version 4.1.2). A heatmap was created to visualize the data matrix using the “heatmap.2” function from the “gplots” package. For the hierarchical clustering, we utilized the “pvclust” function from the “pvclust” package in R (Suzuki and Shimodaira, 2006). The dendrogram was created based on the Euclidean distances between variables and was cut into clusters using the average linkage method. To assess the robustness of the clusters, the “pvclust” function calculated AU (Approximately Unbiased) *p*-values for hierarchical clustering via multiscale bootstrap resampling. We performed 30,000 bootstrap replicates to assess the uncertainty in our hierarchical cluster analysis.

### 2.4. Structure analysis of fungal communities

We analyzed the structure of fungal communities using Picante R package (Kembel et al., 2010). The community phylogenetic and phenotypic over/underdispersion was quantified using NRI and NTI (Salazar et al., 2016). NRI measures the extent to which taxa within a community are closely related to each other than would be expected by chance. NTI measures the extent to which the phylogenetic distance between the closest relatives of co-occurring taxa is greater or smaller than expected by chance. Positive values of NTI and NRI indicate that similar taxa (phylogenetically and phenotypically) co-occur more than expected by chance; negative values indicate that similar taxa are not likely to co-occur; values near 0 indicate random distribution. The randomization to generate null communities was done by shuffling phylogeny and stress tolerance dendrogram taxa labels in order to calculate the standardized effect sizes for NRI and NTI (abundance weighted model, *n* = 1,000 per community; Picante package).

### 2.5. Competition assays for infectivity and stress tolerance

We selected field isolates that exhibited different stress tolerance patterns; SY6, SY16, SY22, SY32, SY98, SY144, SY147, and SY175. The fungal isolates were grown in liquid complete medium for 72 h at 25°C in a shaking incubator at 0.314 rcf. For conidia production, the mycelia were harvested, spread on yeast malt agar plates, and then incubated for 7 days at 25°C under near-UV light (wavelength, 352 nm; Sankyo Denki). The conidia were collected with sterile water, filtered through Miracloth (Millipore), and washed with water again. The conidia were resuspended in a sterile 15% glycerol solution to yield 2 × 10^6^ conidia mL^−1^. The conidia suspension is stored at −80°C until it is ready to be used.

For competition assay for infectivity, the point inoculation method (Lee S. H. et al., 2009) was employed using the wheat cultivar Geumgang. Frozen stocks of conidia suspension were thawed and pooled. The pooled conidia were spin down and resuspended in sterile water to make 1 × 10^6^ conidia mL^−1^. Ten microliters of conidia suspension were injected into the center spikelet of a wheat head at early anthesis. All inoculations were repeated at least 15 times, and the wheat heads were covered with plastic bags for 3 days to maintain humidity conditions. At day 7 and day 14, the samples were harvested, lyophilized, and ground into small particles using a grinder. We used Lysing Matrix S with a Fastprep-24TM 5G instrument (MP biomedicals, Irvine, CA, United States) to lyse cells for the preparation of fungal DNA extraction. Fungal DNA was extracted by using CTAB method (Leslie and Summerell, 2006). The assay was repeated two times.

For competition assay in stress condition, frozen stocks of conidia suspension were thawed and pooled. Two microliters of the conidia suspension were inoculated into 200 μL complete media supplemented with different chemicals on 96-well plates. The chemicals and their concentrations were same as described above. The cultures in the 96-well plates were incubated for 4 days at 25°C in plastic containers to prevent evaporation. To extract fungal DNA, the mycelia were harvested and resuspended in a lysis buffer (2% Triton X-100, 1% SDS, 0.1 M NaCl, 10 mM Tris-Cl, 1 mM EDTA and pH 8.0). We used 0.5 mm Zirconia/Silica beads (BioSpec Products, Bartlesville, OK, United States) with a Fastprep-24TM 5G instrument (MP biomedicals) to lyse cells. The fungal DNA was extracted with a mixture of phenol: chloroform: isoamyl alcohol (25: 24: 1). The assay was repeated two times.

To create an inoculum standard, we inoculated 2 μL of individual conidia stock into a separate well containing 200 μL of complete medium. The cultures on the 96-well plates were incubated for 2 days at 25°C. The fungal cultures were pooled, and DNA was extracted as described above. The extracted fungal DNA was used as an inoculum standard prior to competitive assay.

### 2.6. Estimation of relative abundance of fungal isolates

The relative abundance of each strain in competitive assays was determined by analyzing the relative proportions of their DNA variants (Carr et al., 2009; Seroussi, 2021). The *TEF-1α* sequence was selected because each isolate has a unique single-nucleotide sequence variant (SNV) in the region (Supplementary Figure S3 and Supplementary Data S2). First, The *TEF-1α* region was amplified from the fungal DNA using FgEF-F and FgEF-R primers which are specific to FGSC (Supplementary Table S1). The PCR products were purified and submitted to Bioneer for Sanger sequencing. The *TEF-1α* region was sequenced using two forward primers (FusF and FgEF-F2) and two reverse primers (FgEF-R and FgEF-R2). Relative copy number information of a unique single-nucleotide sequence variant was extracted from the sequencing chromatograms. We calculated the occurrence ratio of SNVs from sequence trace files using the SnapGene software (Dotmatics, Boston, MA, United States). The values of the same condition were averaged. Experiments comparing 8 strains resulted in 8 occurrence ratios of SNV for the pooled inoculum (*I*) and the samples (*S*). Finally, *S*/*I* was calculated for each stress condition and wheat infection; these are presented in Supplementary Data S3.

## 3. Results

### 3.1. Isolation of *F. graminearum* and *F. asiaticum* strains in cereal fields

To determine the population structure of *F. graminearum* and *F. asiaticum*, we isolated 205 strains from diseased cereals crops in 18 sites in southern provinces of the Republic of Korea (Figure 1A and Supplementary Data S1). The samples were from barley (42%), rice (34%), wheat (23%), and oat (1%) (Figure 1B). Sequences of the translation elongation factor-1 α (*TEF-1α*) gene were analyzed for species identification. *F. asiaticum* represents 91% of the isolates while *F. graminearum* accounts for the remaining 9% (Figure 1C). We constructed a phylogenetic tree of the fungal strains based on the *TEF-1α* sequences inferred by maximum-likelihood analysis (Supplementary Figure S1). The phylogenetic analysis with reference strains validated the species identification. A chi-square test was performed to compare the species compositions in different hosts and locations. Surprisingly, the results showed that there was no significant difference in species compositions between hosts and locations (*p* > 0.1). Trichothecene genotypes of the 205 isolates were determined using multiple PCR targeting the *TRI3* and *TRI12* genes (Starkey et al., 2007). Based on trichothecene genotypes, the *F. asiaticum* and *F. graminearum* strains were divided into three chemotype groups: NIV, 3-ADON, and 15-ADON. The predominance of the NIV chemotype in our study is consistent with findings from the previous studies (Lee J. et al., 2009; Ahn et al., 2022; Figure 1C). However, chi-square test showed that there was no significant difference in chemotype compositions between hosts and locations (*p* > 0.1) (Figure 1D). These results contrast to the previous hypothesis that the species and trichothecene chemotypes have host preference (Lee et al., 2010; Xu et al., 2021; Ahn et al., 2022).

### 3.2. High-throughput screening revealed phenotypic diversity of *F. graminearum* and *F. asiaticum* isolates

**Figure 2:**
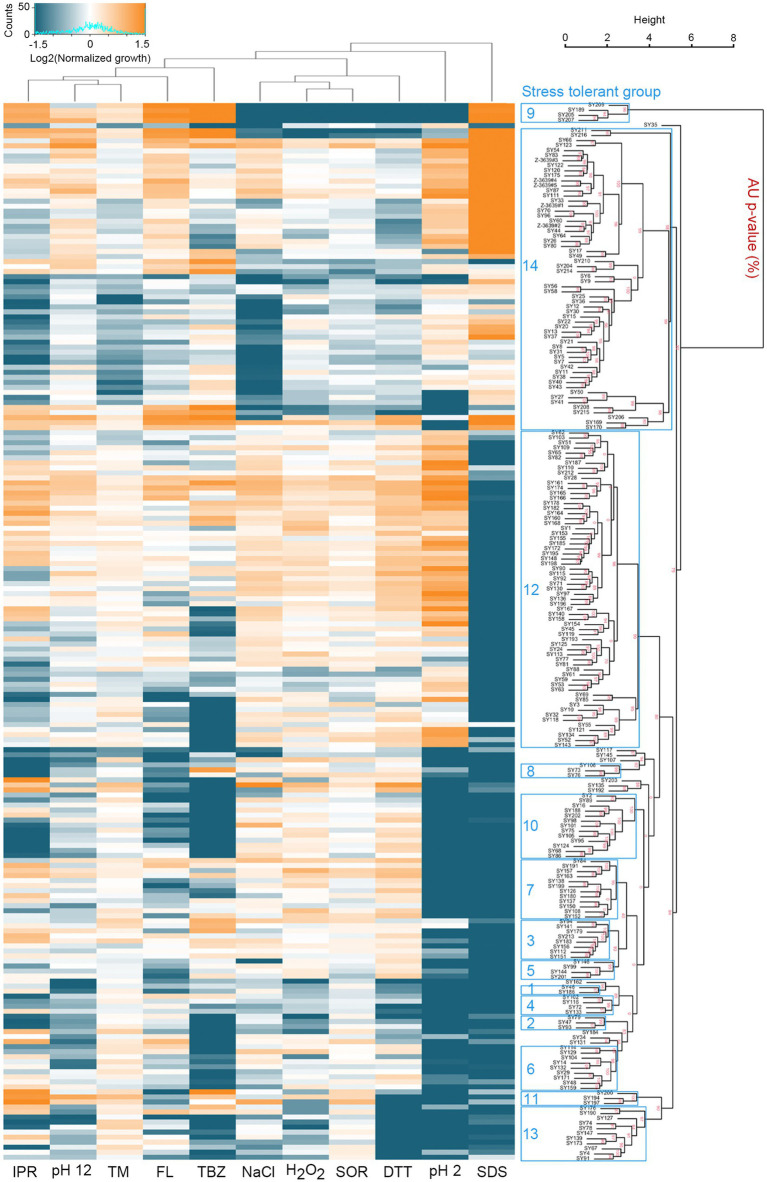
High-throughput phenotypic screening reveals intraspecific variation in stress tolerance. *F. graminearum* and *F. asiaticum* strains were grown on complete media (CM) on 96-well plates. Various chemicals were added to CM to assays their effects on growth by oxidative [0.31 mM hydrogen peroxide (H_2_O_2_)], osmotic [1 M sorbitol (SOR) or 1 M NaCl], cell wall [0.1 mg mL^−1^ sodium dodecyl sulfate (SDS)], fungicide [0.05 μg mL^−1^ fludioxonil (FL), 125 μg mL^−1^ iprodione (IPR), or 0.625 μg mL^−1^ tebuconazole (TBZ)], ER [2 μg mL^−1^ tunicamycin (TM) or 1.25 mM dithiothreitol (DTT)], and acidic (pH 2) or alkaline (pH 12) pH stresses. Each stress condition had two replicate wells per chemical. The cultures in the 96-well plates were incubated for 48 h at 25°C and the OD_600_ value was measured. To compare the growth of the strains under different stress conditions, the growth in CM was used as a reference point and the growth in stress conditions was normalized accordingly. The resulting values were used to calculate the Log2 fold difference from the average growth for heatmap analysis. Supplementary Data S1 contains the values that were used to generate the heatmap. Growth under different stress conditions is shown in a heatmap where orange indicates above-average growth and green-blue indicates below-average growth. Hierarchical clustering of the fungal strains was performed based on their stress tolerance pattern (Suzuki and Shimodaira, 2006). Approximately unbiased (AU) *p*-values for each stress tolerant group were calculated using bootstrap resampling techniques (30,000 replicates). Clusters with AU *p* > 95% are highlighted by blue rectangles, indicating strong support from the data.

We performed a high-throughput screening for chemical stress tolerance. Specifically, we grew fungal strains in complete media supplemented with 11 different chemicals on 96-well plates and compared stress tolerance by measuring the OD_600_ (optical density at the 600 nm wavelength) of the cultures. *F. graminearum* and *F. asiaticum* isolates showed a wide range of variation in stress tolerance (Figure 2 and Supplementary Data S1). Based on their stress tolerance pattern, the strains were classified into 14 groups. Distinct stress tolerance patterns were observed in each group. For instance, strains in group 9 exhibited tolerances to fungicides such as iprodione, fludioxonil, and tebuconazole. Groups 9 and 14 were tolerant to sodium dodecyl sulfate (SDS), while other groups were sensitive to SDS. In an acidic condition (pH 2), groups 12 and 14 were tolerated but other groups displayed strong sensitivity. Interestingly, *F. graminearum* strains had similar phenotypes and were categorized into group 14. *F. asiaticum* strains, despite being the same species, exhibited greater diversity and were classified into multiple groups. Correlation coefficients were calculated to determine the relationships among the 11 stress tolerance traits (Supplementary Figure S2). A strong positive correlation (*R* = 0.78) was observed between dithiothreitol and hydrogen peroxide tolerances. Additionally, the tolerance to osmotic stress induced by 1 M sorbitol showed a weak correlation (*R* = 0.66) with either hydrogen peroxide or sodium chloride.

### 3.3. Phenotypic clustering of *F. graminearum* and *F. asiaticum* communities

**Figure 3:**
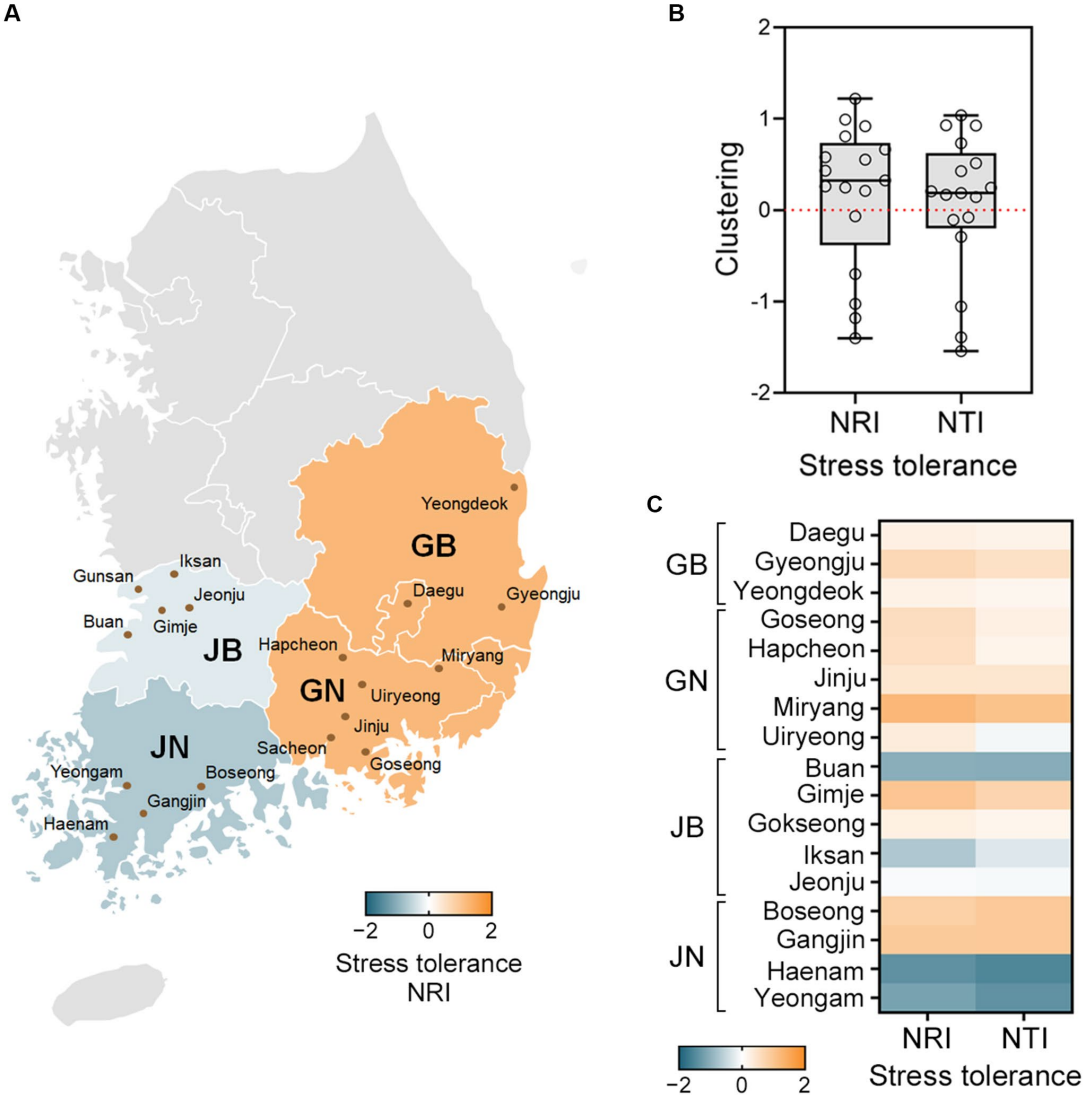
Communities of *F. graminearum* and *F. asiaticum* exhibit phenotypic clustering in certain local regions. Net Relatedness Index (NRI) and Nearest Taxon Index (NTI) values were calculated based on distance matrices of the stress tolerant groups. **(A)** The NRI and NTI values above 0 indicate clustering (orange) and values below 0 indicate overdispersion (green-blue). The NRI values for each province are shown in a heatmap. GB and GN showed phenotypic clustering, while JB and JN showed overdispersion. GB, Gyeongsangbuk-do; GN, Gyeongsangnam-do; JB, Jeollabuk-do; JN, Jeollanam-do. **(B)** Local tests for phenotypic structure of the fungal strains. The NRI and NTI values are represented using a box and whisker plot, with the box covering 50% of the data, the vertical bars showing the full data range, and the middle box line indicating the group median. **(C)** The NRI and NTI values for each site are shown in a heatmap. In both the GB and GN provinces, all sites exhibited NRI and NTI values above 0 (orange), indicating phenotypic clustering. The NRI and NTI values varied in the JB and JN provinces, and some sites exhibited overdispersion (green-blue).

Structure analysis of the fungal communities revealed that fungal strains are more closely related in certain local regions (Figure 3). Net Relatedness Index (NRI) and Nearest Taxon Index (NTI) values were calculated based on stress tolerant distance matrices (Kembel et al., 2010; Salazar et al., 2016). The NRI values indicate that Gyeongsangbuk-do (GB) and Gyeongsangnam-do (GN) showed phenotypic clustering (NRI > 0), while Jeollabuk-do (JB) and Jeollanam-do (JN) showed overdispersion (NRI < 0) (Figure 3A). Similar trends were observed in the NTI values. Examining the local sites, we found that *F. graminearum* and *F. asiaticum* communities were clustered in most sites (Figures 3B,C). Furthermore, the fungal communities in all GB and GN sites exhibited phenotypic clustering based on stress tolerance (NRI > 0 and NTI > 0). In contrast, JB and JN displayed diverse fungal community structures (Figure 3C). GB and GN, which are geographically close, are often grouped as Gyeongsang-do (G), while JB and JN, also geographically close, belong to Jeolla-do (J). These two regions are separated by high mountains and have distinct climates. Jeolla-do (J) experiences higher annual precipitation and more winter snow compared to Gyeongsang-do (G) (Administration, 2022). It is likely that these geographical and climate differences contribute to the observed variations in fungal community structures (Backhouse, 2014; Scala et al., 2016; Xu et al., 2021; Wang et al., 2022).

### 3.4. The diversity of the fungal community decreased under chemical stress and host infection

**Figure 4:**
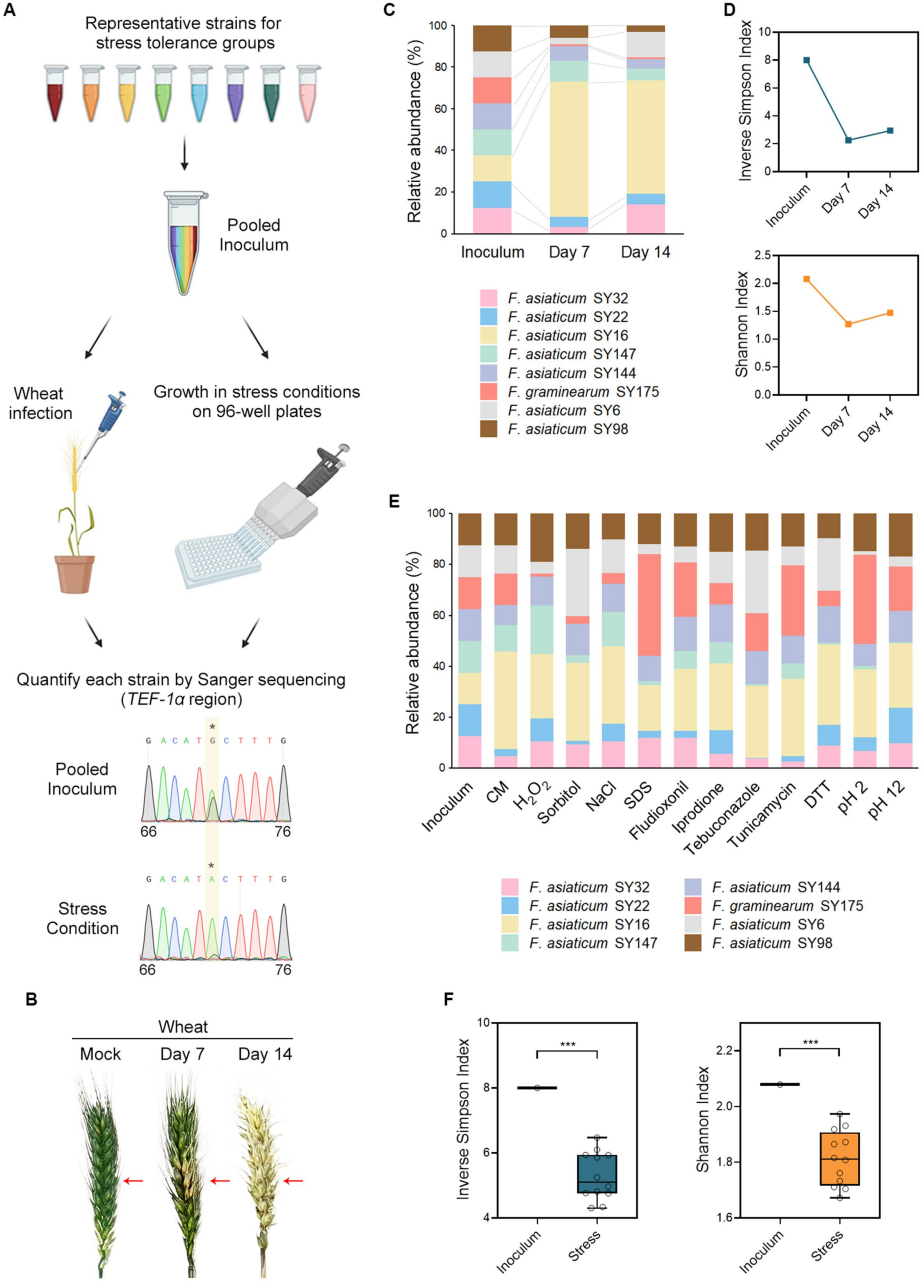
Wheat infection and exposure to chemical stresses decrease the diversity of the fungal community. **(A)** Experimental scheme of competition assays for infectivity and stress tolerance. We selected *F. graminearum* and *F. asiaticum* strains that represent different stress tolerance patterns; SY6, SY16, SY22, SY32, SY98, SY144, SY147, and SY175. Equal amounts of freshly harvested conidia of each strain were mixed and the resulting mixtures were used to inoculate wheat heads by injection or grow under stress conditions in 96-well plates. Fungal DNA was isolated from the wheat heads or 96-well cultures for sequencing. The *TEF-1α* region was amplified from the fungal DNA and sequenced using Sanger sequencing. Relative copy number information of a unique single-nucleotide sequence variant was extracted from the sequencing chromatograms. For example, SY147 has an A to G variant at nucleotide position 71 that was unique to the strain. The A to G ratio decreased from 0.417 in the pooled inoculum to 0.078 in a certain stress condition, indicating 82% reduction in the relative abundance of the strain. **(B)** The disease symptoms of infected wheat heads. The pooled inoculum was injected into the center spikelet of a wheat head at early anthesis. As a mock control, the wheat head was inoculated with sterile water. The injection point is indicated by the red arrows. Photographs were taken on days 7 and 14 to monitor the development of the disease. **(C)** Relative abundance of the fungal strains during the wheat infection. The colors in the graph represent the pooled strains, which were equal in abundance in the inoculum. On day 7, *F. asiaticum* SY16 emerged as the dominant strain, while *F. graminearum* SY175 decreased in abundance. **(D)** Based on the relative abundance of fungal strains, we calculated the inverse Simpson and Shannon indices. The decrease in these alpha diversity indices on days 7 and 14 suggests a sharp decline in the diversity of the fungal community during wheat infection. **(E)** The abundance patterns of fungal strains varied across different stress conditions. Each bar in the graph shows the strain composition of a particular stress condition 4 days after inoculation. The different colors in the graph represent the pooled strains, which had equal abundance in the inoculum. CM, complete medium only; H_2_O_2_, hydrogen peroxide; NaCl, sodium chloride; SDS, sodium dodecyl sulfate; DTT, dithiothreitol. **(F)** The Inverse Simpson and Shannon indices were determined for each stress condition based on the relative abundance of fungal strains, and the results are presented using box and whisker plots. The box spans the interquartile range, the vertical lines show the full range of data, and the middle line within the box represents the median value. The presence of asterisks indicates a significant decline in the average alpha diversity indices compared to the initial inoculum (*p* < 0.001), implying that the diversity of the fungal community decreased in stress conditions.

To investigate the cause of clustering, competition assays were conducted under chemical stress and host infection (Figure 4A). A pooled inoculum of eight field isolates, each possessing distinct stress tolerance traits, was injected into wheat heads, resulting in typical symptoms of FHB (Figure 4B). DNA was extracted from infected wheat heads harvested at days 7 and 14, and the *TEF-1α* region was sequenced. The relative abundance of fungal strains during wheat infection was estimated from the sequencing chromatograms (Figures 4A,C). Interestingly, *F. asiaticum* SY16 (NIV type) emerged as the dominant strain, while *F. graminearum* SY175 (DON type) showed a decrease in abundance. Inverse Simpson and Shannon indices dropped on days 7 and 14, indicating a sharp decline in the diversity of the fungal community during wheat infection (Figure 4D). This suggests that competition in the host results in overrepresentation of infectivity traits leading to clustered communities. In parallel, the pooled inoculum was grown in various stress conditions such as oxidative stress, osmotic stress, cell wall stress, fungicide stress, ER stress, and pH stress, resulting in diverse abundance patterns across different stress conditions (Figure 4E). For example, *F. graminearum* SY175 showed tolerance in SDS and pH 2 when grown in single culture (Figure 2 and Supplementary Data S1). When grown in mixed cultures under the same stress conditions, this strain was more competitive and became predominant (Figure 4E). *F. asiaticum* SY16 showed a highly competitive ability in the mixed cultures when grown in complete medium and stress conditions. This observation is consistent with the high infectivity of *F. asiaticum* SY16 in wheat heads (Figure 4C). To assess the impact of stress on fungal diversity, we calculated the Inverse Simpson and Shannon indices for each stress condition. The results showed that the diversity indices in stress conditions were significantly lower than in the initial inoculum (Figure 4F). This suggests that exposure to stress leads to an overrepresentation of stress-tolerant traits, reducing the diversity in the pool. Overall, our lab experiments demonstrated that fungal diversity decreased in both wheat infection and stress conditions.

## 4. Discussion

The findings of our study provide evidence supporting the idea that infectivity and stress tolerance traits are significant drivers of clustering in communities of plant pathogenic fungi. High-throughput screening revealed that the *F. graminearum* and *F. asiaticum* isolates had a wide range of phenotypes. We also observed interesting patterns of phenotypic clustering in the local sites. In competition assays, a few strains were able to dominate the fungal communities, leading to decreased diversity in the pool. This phenomenon was particularly evident in the case of wheat infection, where a *F. asiaticum* single strain became predominant in the middle of the disease’s progression.

**Figure 5:**
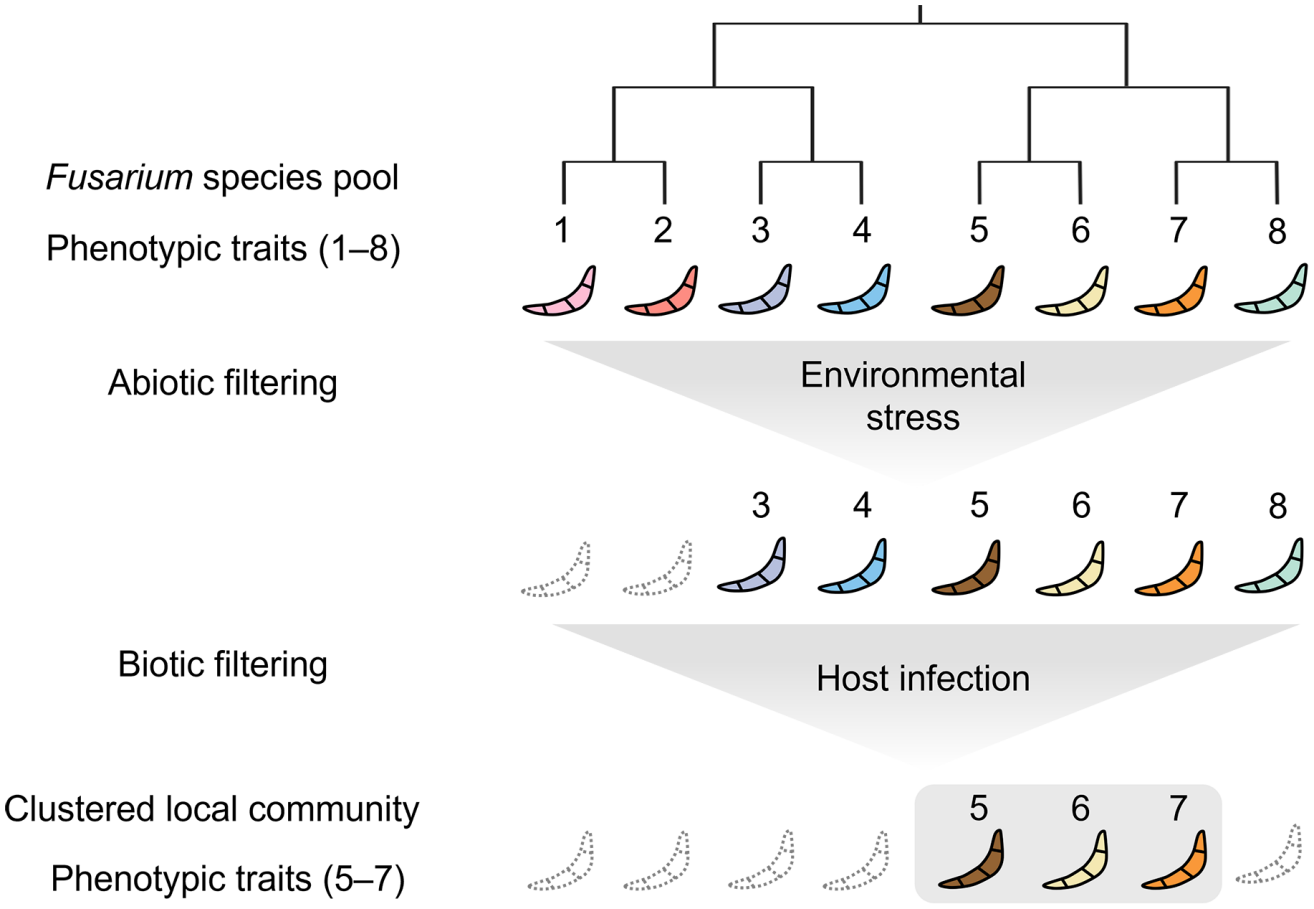
Conceptual representation of the clustering model tested for *F. graminearum* and *F. asiaticum* strains of the Republic of Korea. Fungal strains from the Republic of Korea *Fusarium* species pool are subjected to an abiotic filter that selects for strains with tolerances to various environmental stresses, such as desiccation, harsh temperature, and agrochemicals. The selected strains then segregate into local communities according to local biotic filters, such as host infection, which leads to a clustered phenotypic structure. As a result, the local communities have fewer phenotypic traits and reduced phylogenetic diversity. In summary, abiotic stresses and biotic interactions generate phenotypic and phylogenetic clustering in the plant pathogenic fungi.

These observations fit well with the modern coexistence theory (Mayfield and Levine, 2010) that competition operates by competitive ability differences, leading to the exclusion of distantly related taxa and thus to phenotypic clustering. Abiotic filtering also results in the overrepresentation of environmental tolerance traits leading to phenotypically clustered (related) communities. Based on this theory, we propose a mechanism by which both abiotic and biotic filtering have shaped the communities of *F. graminearum* and *F. asiaticum* (Figure 5). *F. asiaticum* was the dominant species in the field, whereas *F. graminearum* was less abundant. The geographical distribution of these two species was related to the climate, with *F. graminearum* predominant in the cold temperate region and *F. asiaticum* in the warm temperate region of East Asia (Suga et al., 2008; Lee et al., 2010; Wang et al., 2022). This pattern is consistent with a previous observation that climate plays a significant role in shaping the distribution of various *Fusarium* species responsible for FHB globally (Backhouse, 2014; Xu et al., 2021). By infecting the host plant, the plant pathogens gain access to resources and outcompete other microorganisms that lack the ability to infect plants. Biotic filtering, resulting from competition for host infection, leads to the overrepresentation of infectivity-related traits, generating phenotypic clustering. Our study demonstrated that competition between *Fusarium* strains for infection and colonization of host plants resulted in the predominance of a highly infectious strain (Figure 4C). Previous greenhouse and field studies on other plant pathogenic fungi, has underscored the significant role the host plays in determining the outcome of such competitions (Sommerhalder et al., 2011; Zhan and McDonald, 2013; Bernasconi et al., 2022). Wang et al. showed that a single wheat head in the field is colonized by a single dominant species: *F. graminearum* or *F. asiaticum* (Wang et al., 2022). Similarly, most wheat heads with FHB in Europe were colonized by a single *Fusarium* species: *Fusarium graminearum* or *Fusarium culmorum* (Klix et al., 2008). The use of monoculture and crop rotation resulted in the homogeneous distribution of host plants, which increased the competition between *Fusarium* strains and selected for few strains with the ability to infect a wide range of crop plants. As a result, agricultural intensification resulted in locally clustered communities of plant pathogenic fungi in the field.

Large-scale phenotyping has been implemented to assess the phenotypic variations of many field isolates (Talas et al., 2016; Dutta et al., 2021). However, the filamentous growth of these fungi makes it difficult to use the high-throughput approach. Measuring the radius of a colony on agar plates is often laborious and does not provide a quantitative measure of growth. In this study, we utilized the high-throughput approach that developed to efficiently examine the tolerance of the yeast-like fungi to various stressors, including antifungal agents (Siles et al., 2013; Caplan et al., 2020; Aguiar-Cervera et al., 2021; Du Bois et al., 2022). To achieve this, a suspension conidia (asexual spores) was used to inoculate liquid media on 96-well plates. This method allowed for easy control of the inoculum size and scalability of experiments. By using small volumes of media for 96-well cultures, toxic chemical usage was reduced, and quantitative measurement of growth as OD using a plate reader saved time and reduced human bias. However, it should be noted that chemicals with colors could not be used due to their interference with OD readings, presenting a potential limitation. Our approach can be used for the identification of antifungal resistant strains in the field and for screening mutant libraries in various stress conditions. We believe our high-throughput screening approach will be a valuable toolkit for study of filamentous fungi.

One interesting observation from our high-throughput screening was that stress tolerance traits had a wide range of phenotypic variation *F. asiaticum* (Figure 3A). Similarly, global strains of the fungal wheat pathogen *Zymoseptoria tritici* showed a high degree of phenotypic diversity across all measured traits. This indicates rapid and divergent evolution of these traits among related taxa (Goberna et al., 2014). This rapid evolution of stress tolerance traits may be due to genome plasticity, which many pathogenic fungi use to adapt to environmental and host stress (Covo, 2020; Bussotti et al., 2021; Min et al., 2021; Smith et al., 2022). For instances, in the human pathogen *Candida albicans*, genetic changes and aneuploidy are frequently detected, and are known to contribute to antifungal resistance, stress tolerance, and virulence (Legrand et al., 2019; Min et al., 2021; Smith et al., 2022). In the context of *Fusarium graminearum*, population genomics analyses have revealed a high level of genetic diversity and evidence of extensive recombination within the population (Talas and McDonald, 2015; Talas et al., 2016). These findings underscore the dynamic nature of pathogenic fungal genomes and their inherent capacity to adapt and evolve in response to environmental and host-induced stresses. This diversity and plasticity potentially play a crucial role in their ability to cause disease and thrive in various environments, thereby posing significant challenges to disease management strategies.

In conclusion, we propose a model for *Fusarium* community assembly (Figure 5). Our analysis of community structure reveals that *F. graminearum* and *F. asiaticum* evolve stress tolerance traits rapidly and divergently. However, abiotic and biotic filtering result in overrepresentation of certain traits, leading to phenotypically clustered communities. These findings have important implications for understanding the dynamics of fungal communities in agriculture. By highlighting the role of infectivity and stress tolerance traits in driving clustering, our study provides insights into the factors that shape the structure and composition of pathogenic fungi communities. Future studies focusing on the genetic basis of these traits using high-throughput screening and multi-omics approaches will further elucidate the molecular mechanisms underlying community assembly in pathogenic fungi.

## Data availability statement

The data presented in the study are deposited in the NCBI repository, accession numbers from OR348436 to OR348639.

## Author contributions

SC, JWY, HS, and KM conceived and designed the experiments. SC, JWY, HJ, SS, DW, LSK, and KM conducted the experiments. HS and KM supervised the experiments. SC and KM performed statistical analysis and interpreted the data. SC, JWY, HS, and KM wrote and edited the manuscript. All authors read and approved the final manuscript.

## Funding

This work was supported by the National Research Foundation of Korea (2022R1I1A1A01065138 and 2021R1C1C1004200) and the project PJ015741022023 of National Institute of Crop Science, Rural Development Administration, Republic of Korea.

## Conflict of interest

The authors declare that the research was conducted in the absence of any commercial or financial relationships that could be construed as a potential conflict of interest.

## Publisher’s note

All claims expressed in this article are solely those of the authors and do not necessarily represent those of their affiliated organizations, or those of the publisher, the editors and the reviewers. Any product that may be evaluated in this article, or claim that may be made by its manufacturer, is not guaranteed or endorsed by the publisher.

## Footnotes

^1^ https://ncbi.nlm.nih.gov/popset/12003438
