# Supplementary material for: Infectivity and stress tolerance traits affect community assembly of plant pathogenic fungi
Source: Front Microbiol. 2023 Aug 25;14:1234724. doi: 10.3389/fmicb.2023.1234724 (PMC10486888; doi:10.3389/fmicb.2023.1234724)
Supplement: Supplementary file 1 [file Data_Sheet_1.PDF]

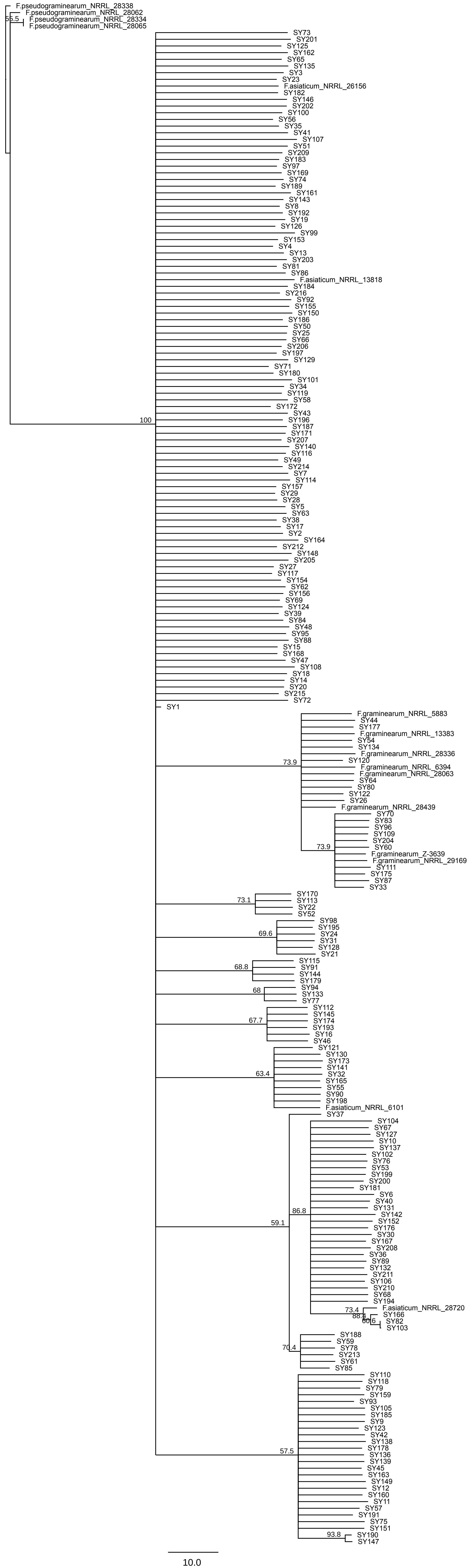
F.pseudograminearum_NRRL_28338
F.pseudograminearum_NRRL_28062
55.5
F.pseudograminearum_NRRL_28334
F.pseudograminearum_NRRL_28065
100
F.asiaticum_NRRL_26156
F.asiaticum_NRRL_13818
SY1
F.graminearum_NRRL_5883
F.graminearum_NRRL_13383
F.graminearum_NRRL_28336
F.graminearum_NRRL_6394
F.graminearum_NRRL_28063
F.graminearum_NRRL_28439
F.graminearum_Z-3639
F.graminearum_NRRL_29169
73.9
73.1
69.6
68.8
68
67.7
63.4
F.asiaticum_NRRL_6101
86.8
59.1
F.asiaticum_NRRL_28720
73.4
70.4
57.5
93.8
10.0

Supplementary Figure 1. Full phylogenetic tree of 205 strains of *F. graminearum* and *F. asiaticum.* The phylogeny was based on the sequences of the translation elongation factor-1 α (*TEF-1α*) gene inferred by maximum-likelihood (ML) analysis. The ML analysis was performed utilizing the General Time Reversible model with Invariant sites and Gamma distribution (GTR+I+G). Bootstrap analysis with 1000 replicates was performed, and the resulting percentages were displayed on the corresponding branches of the tree. The *F. graminearum* and *F. asiaticum* strains are divided into separate groups. *F. pseudograminearum* reference strains were set as an outgroup. Reference sequences were obtained from NCBI PopSet (https://ncbi.nlm.nih.gov/popset/12003438).

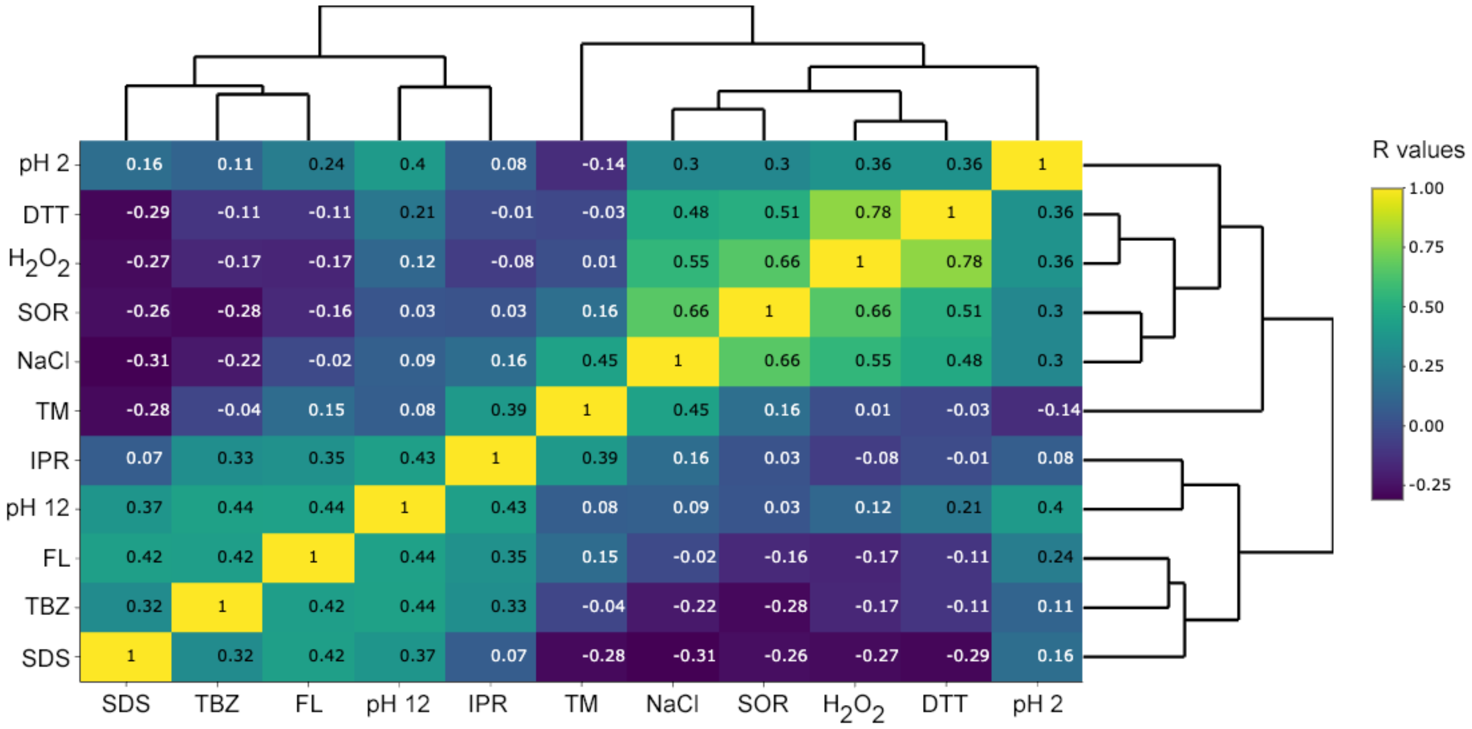

Supplementary Figure 2. Correlation coefficients among 11 stress tolerance traits measured on 96-well plates. Correlation coefficients among the 11 traits were estimated using the Log2 fold difference from the average growth. Positive correlation coefficients (R values) were represented by yellow, while negative correlation coefficients were represented by blue. To assess the stress tolerance traits, various chemicals were tested on 96-well plates. SDS, sodium dodecyl sulfate; TBZ, tebuconazole; FL, fludioxonil; pH 12, alkaline pH; IPR, iprodione; TM, tunicamycin; NaCl, sodium chloride; SOR, sorbitol; H_2O_2, hydrogen peroxide; DTT, dithiothreitol; pH 2, acidic pH.

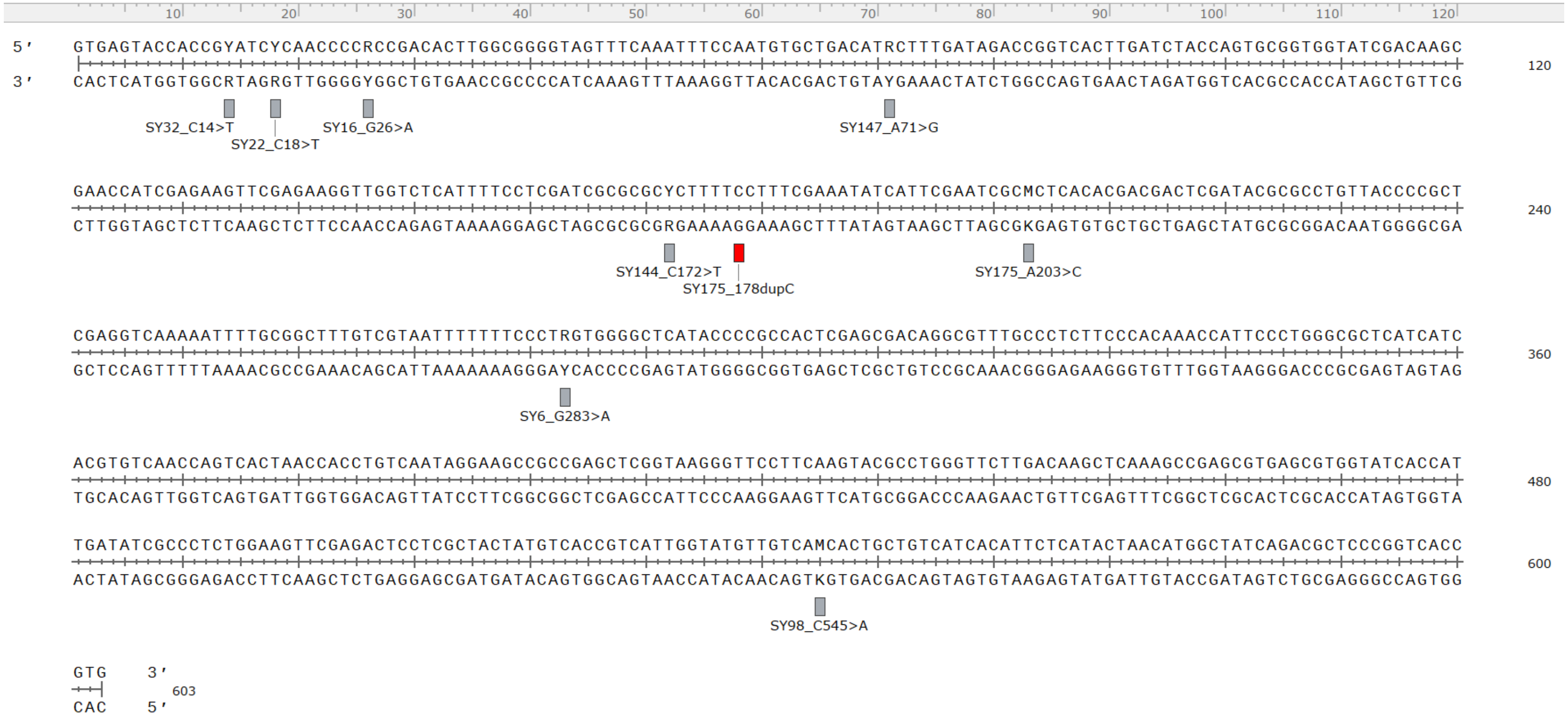

Supplementary Figure 3. Single-nucleotide sequence variants (SNVs) in the *TEF-1α* region. This sequence map shows internal DNA sequence of *TEF-1α* and SNVs. A box indicates the location of SNV for the indicated strain. DNA sequence change was described after the strain name.

Supplementary Table 1. Oligonucleotides used in this study.

Name	Sequence (5' to 3')	Description	Reference
FusF	TGGGTAARGAGGASAAGACTCACC	Fusarium-specific forward primer for sequencing *TEF1α* region	Hafez et al. (2020)
EF-2	GGARGTACCAGTSATCATGTT	Fusarium-specific reverse primer for sequencing *TEF1α* region	O’Donnell et al. (1998)
3CON	TGGCAAAGACTGGTTCAC	Genotype PCR forward primer for detecting *TRI3* allele	Starkey et al. (2007)
3NA	GTGCACAGAATATACGAGC	Genotype PCR reverse primer for detecting *TRI3* allele (NIV-type specific)	Starkey et al. (2007)
3D15A	ACTGACCCAAGCTGCCATC	Genotype PCR reverse primer for detecting *TRI3* allele (15-ADON-type specific)	Starkey et al. (2007)
3D3A	CGCATTGGCTAACACATG	Genotype PCR reverse primer for detecting *TRI3* allele (3-ADON-type specific)	Starkey et al. (2007)
12CON	CATGAGCATGGTGATGTC	Genotype PCR forward primer for detecting *TRI12* allele	Starkey et al. (2007)
12NF	TCTCCTCGTTGTATCTGG	Genotype PCR reverse primer for detecting *TRI12* allele (NIV-type specific)	Starkey et al. (2007)
12-15F	TACAGCGGTCGCAACTTC	Genotype PCR reverse primer for detecting *TRI12* allele (15-ADON-type specific)	Starkey et al. (2007)
12-3F	CTTTGGCAAGCCCGTGCA	Genotype PCR reverse primer for detecting *TRI12* allele (3-ADON-type specific)	Starkey et al. (2007)
FgEF1-F	TCTGTACGTACGACCCTTCA	FGSC-specific forward primer for sequencing *TEF1α* region	This study
FgEF1-R	GGAAGTACCAGTGATCATGTT	FGSC-specific reverse primer for sequencing *TEF1α* region	This study
FgEF1-F2	ATGGGTAAGGAGGAGAAGAC	FGSC-specific forward primer for sequencing *TEF1α* region	This study
FgEF1-R2	GAGCGTCTGATAGCCATGTT	FGSC-specific reverse primer for sequencing *TEF1α* region	This study

Supplementary Note 1. Press Release - Fusarium Outbreak in South Korea (English translation). In 2021, the Rural Development Administration of South Korea issued a warning to farmers in barley, oat, and wheat-growing areas to be vigilant about pre- and post-harvest management in order to minimize damage from the increased occurrence of Fusarium Head Blight (FHB). The spread of FHB has been worsened by heavy rainfall in May 2021. The press release was obtained from the Rural Development Administration's website in South Korea (the link can be found in the References). The original version was written in Korean and has been translated into English.

Rural Development Administration	**Press Release**	보다 나은 농촌진흥청<br>대한민국 대전환 한국판뉴딜

Please cooperate to release the following information simultaneously with distribution.

Distribution date	May 28, 2021 (5 pages total)	Responsible department	Food Industry Technology Team<br>Harmful Organism Team<br>Crop Infrastructure and Cultivation Environment Team
Responsible Manager	Jeong-hwa Kim, Manager (063-238-1040)<br>Byung-woo Jung, Team Leader (063-238-1495)<br>Ik-seong Jeon, Team Leader (063-238-3391)<br>Jung-kyung Moon, Manager (063-238-5300)<br>Tae-hwan Noh, Manager (031-695-0630)	Responsible Person	Woo-il Lee, Instructor (063-238-1046)<br>Yoon-hee Choi, Manager (063-238-1499)<br>Jeom-sun Kim, Researcher (063-238-3399)<br>Sang-min Kim, Researcher (063-238-5344)<br>Jung-Wook Yang, Researcher (031-695-0651)

Prevention of Fusarium Head Blight, important before and after harvest

- High risk of spread due to frequent rainfall in May

- Harvest on clear and dry days, store in low temperature and dry environment.

□ The Rural Development Administration (Director, Tae-woong Heo) has urged special attention to be paid to pre- and post-harvest management in the major barley, oat, and wheat growing areas* of Jeonbuk, Jeonnam, and Gyeongnam provinces to minimize damage from the increased occurrence of Fusarium Head Blight (FHB) compared to last year, which has spread due to the frequent rainfall.

* 2020-2021 cultivated area: Jeonbuk 31.4%, Jeonnam 43.9%, Gyeongnam 8.9% (expressed as the percentage of the total cultivated area)

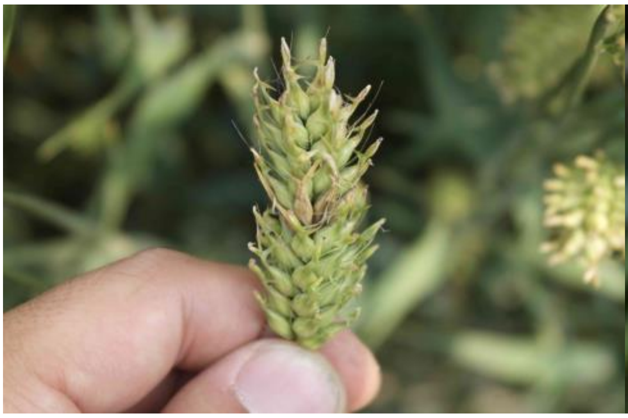

< Fusarium Head Blight symptoms in barley>

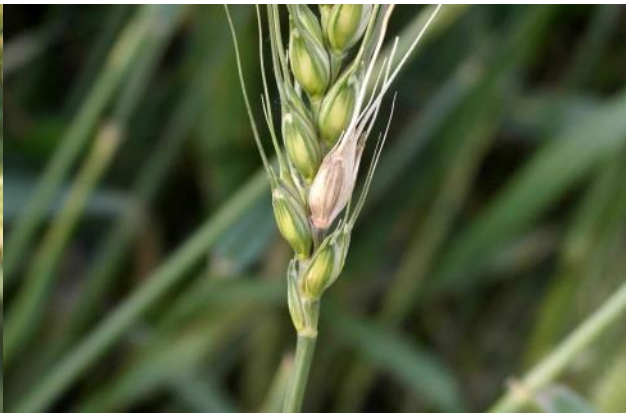

< Fusarium Head Blight symptoms in wheat>

○ According to a survey of the macadamia-growing areas in Jeonju, Iksan, Gunsan, Gimje, Buan, Jeonbuk, Yeongam, Haenam, Gangjin, and Boseong in Jeonnam, Sachon, Goseong, Jinju, Uiryeong, and Hapcheon in Gyeongnam, the percentage of diseased ears was 5.6%, 3.4%, and 10.5%, respectively, higher than 0.2%, 0.5%, and 3.2% in 2020.

**Region**	**% of diseased ears with Red Fusarium Head Blight**				
	2017	2018	2019	2020	**2021**
**Jeonbuk**	0.4	24.4	0.2	0.2	**5.6**
**Jeonnam**	0.4	27.3	0.3	0.5	**3.4**
**Gyeongnam**	1.0	8.4	0.1	3.2	**10.5**

National Academy of Agricultural Science FHB Survey, Jung-Wook Yang, Agricultural Researcher

□ Fusarium Head Blight generally occurs between mid-to-late April when the ears appear, and during the early stages of maturity when the grains are soft (milk stage), it is easy to develop the disease if warm and humid weather with continuous rainfall continues for 2-3 days, so spraying with pesticides can prevent the disease.

○ This year, there is concern among farmers that the quality of crops such as barley may be affected by disease outbreaks due to increased rainfall from mid-May. 'Scouting' can help predict the future occurrence of disease by considering factors such as the density of pests and diseases, current occurrence, crop growth conditions, and weather forecasts.

□ As the harvest of these crops begins in early June, it is important for farmers to monitor their fields for signs of disease and take appropriate measures to minimize damage.

* It is important to predict the future occurrence of disease by considering factors such as the density of

pests and diseases, current occurrence, crop growth conditions, and weather forecasts.

○ For crops such as barley and oats, which are still within the safe use period of certain pesticides, farmers can apply these to prevent disease before the harvest.

○ However, for crops such as wheat, which are close to harvest, alternative methods such as drying the fields may be necessary to prevent the growth of mold and other diseases.

○ Once harvested, crops must be immediately dried and stored properly to prevent the growth of mold and fungus.

* The safe moisture content for stored crops varies by type, with barley at 14% (13% for brewing barley) and wheat at 13% for long-term storage.

- Dry grains should be sorted to remove infected grains, and during storage, the indoor temperature should be kept at 15℃ and the relative humidity should be maintained below 65% to inhibit the growth of mold.

□ The Rural Development Administration's Crop Foundation and Director Moon Jung-Kyung urged farmers to dry their crops after harvest and to store them at low temperatures below 15°C with relative humidity below 65%, to prevent the growth of red mold fungus, which is known to thrive in temperatures between 15-25°C and in seed moisture content of over 17%.

□ Kim Dae-nam, a farmer in Buyeo, Chungnam province, said he is paying close attention to managing his fields and crop maintenance in preparation for the upcoming wheat harvest to produce high-quality, safe grain.

□ Information about the safe use and registration of pesticides for the control of red mold fungus in crops such as barley and oats is available on the Rural Development Administration's Nongsaro website (www.nongsaro.go.kr) or the Pesticide Safety Information System (http://psis.rda.go.kr).

OPEN 출처표시 상업용금지 공공누리 공공저작물 자유이용허락

If you need more information or want to cover this news, please contact Kim Sang-min, an agricultural researcher at the Rural Development Administration's Crop Foundation Department, at 063-238-5344.
